# Depressive symptoms influence progression to total knee arthroplasty in patients with early knee osteoarthritis: Evidence from the Osteoarthritis Initiative database

**DOI:** 10.1002/jeo2.70355

**Published:** 2025-07-13

**Authors:** Alessandro Bensa, Luca Bianco Prevot, Andrea Piano, Giuseppe M. Peretti, Giuseppe Filardo

**Affiliations:** ^1^ Service of Orthopaedics and Traumatology Department of Surgery, EOC Lugano Switzerland; ^2^ Università della Svizzera Italiana, Faculty of Biomedical Sciences Lugano Switzerland; ^3^ Residency Program in Orthopedics and Traumatology University of Milan Milan Italy; ^4^ IRCCS Ospedale Galeazzi ‐ S. Ambrogio Milan Italy; ^5^ Department of Biomedical Sciences for Health University of Milan Milan Italy

**Keywords:** depression, OA, OAI, TKA

## Abstract

**Purpose:**

Several factors can influence progression to total knee arthroplasty (TKA) in knee osteoarthritis (OA) patients. Among these, psychological aspects can play a relevant role. The aim of this study was to quantify the influence of depressive symptoms on the progression to TKA in a large population of patients affected by knee OA.

**Methods:**

A total of 912 patients were selected from the Osteoarthritis Initiative (OAI) database. The data collected included demographic data, Kellgren–Lawrence (KL) OA grade, Visual analogue scale (VAS) for pain, Center for Epidemiologic Studies Depression Scale (CES‐D) and the number of patients progressing to TKA. Patients with KL 2, 3 or 4 and with symptomatic knee OA (VAS ≥ 3) were included and followed up to 9 years.

**Results:**

At 9 years, 22.9% of patients underwent TKA. Overall, no significant differences of CES‐D were found between patients who progressed to TKA and patients who did not. However, in patients with KL grade 2, those who progressed to TKA had a significantly higher CES‐D compared to those who did not (6.9 ± 6.6 vs. 5.4 ± 5.2, *p* = 0.018) and the CES‐D score significantly associated with the risk of undergoing TKA (hazard ratio = 1.042, *p* = 0.015). The pairwise comparison of KL 2 patients showed a significant difference in terms of progression to TKA between patients having a CES‐D > 6 and patients having a CES‐D ≤ 6 (*p* = 0.001). None of these differences were statistically significant in the KL 3 and 4 groups.

**Conclusion:**

This study showed that even minor psychological symptoms related to the depression spectrum, well below the threshold of what commonly considered the level at risk for developing clinical depression, are significantly correlated with the trajectory towards TKA in knee OA patients. However, this correlation was observed only in early KL 2 knee OA patients, but not in the more advanced KL 3 and 4 stages of the disease.

**Level of Evidence:**

Level III.

**Abbreviations**
BMI: body mass index
CES‐D: Center for Epidemiologic Studies Depression Scale
CI: confidence interval
HR: hazard ratio
KL: Kellgren–Lawrence
OA: osteoarthritis
OAI: Osteoarthritis Initiative
TKA: total knee arthroplasty
VAS: visual analogue scale

## INTRODUCTION

Knee osteoarthritis (OA) is one of the most common orthopaedic diseases and represents a major cause of knee pain and disability in the adult population [5, 20]. Progressive joint degeneration and symptomatic worsening often lead to the need for unicompartmental or even total knee arthroplasty (TKA) [7, 9, 26]. However, the outcomes of TKA are not always optimal, with up to 20% of patients reporting dissatisfaction due to persisting or recurring symptoms, and recent evidence raised concerns towards the growing number of younger patients receiving TKA indications and the inherent risks [4, 6, 15]. Addressing the factors leading to the disease progression is therefore paramount, to target them and postpone the need for TKA when possible [3, 25].

Several factors can play a role and influence progression to TKA in patients affected by knee OA [16, 18]. Among these, psychological factors and particularly depressive symptoms were proven to be a key aspect. In fact, depression is prevalent in individuals presenting characteristics that are particularly common in knee OA, such as older age, female gender and decreased mobility [14, 24, 29]. Moreover, a higher rate of depression in patients undergoing TKA is well documented [21]. However, previous evidence addressing this crucial factor reported controversial findings, with the literature presenting small cohorts and lacking longitudinal perspective, with relatively short follow‐ups [1]. In this scenario, a large study with long‐term evaluation investigating the impact of depressive symptoms on progression to TKA would be of clinical relevance by raising awareness on their potential role in knee OA patients. The hypothesis of this study is that depressive symptoms could impact the trajectory towards TKA in patients affected by knee OA.

The aim of this study was to quantify the influence of depressive symptoms on the progression to TKA in a large population of knee OA patients.

## MATERIALS AND METHODS

### Study design, patients selection and evaluation

The participants included in this study were selected from the Osteoarthritis Initiative (OAI), a prospective, multicentre, longitudinal, observational research open access project whose primary objective is to explore knee OA natural history and the risk factors associated with its progression. Data used in this study are publicly available at http://www.oai.ucsf.edu/. The OAI study was approved by the Institutional Review Board and Ethics Committee, and all participants provided informed consent before enrollment. The OAI database provides comprehensive data, including radiographic images, clinical information and questionnaires assessing pain and function, as well as psychological status. A Python 3.9‐based algorithm using the Pandas library was used to identify patients meeting the following inclusion criteria:

- Radiographically confirmed knee OA classified as Kellgren–Lawrence (KL) grade 2, 3 or 4.
- Presence of symptomatic knee OA with a visual analogue scale (VAS) for pain ≥3 (in cases where both knees were symptomatic, only the knee with the highest VAS score was considered).
- Follow‐up of 9 years.

For each participant, the following baseline data were collected: age, sex, body mass index (BMI), KL grade, VAS pain (0–10) and the Center for Epidemiologic Studies Depression Scale (CES‐D) score (0–60). Patients were followed up for 9 years documenting whether they underwent TKA during this period.

Knee pain was assessed at baseline using the VAS pain. The VAS pain is a scale ranging from 0 (no pain) to 10 (worst pain imaginable), with participants being asked to report their worst knee pain in the past week [2]. Depressive symptoms were assessed at baseline using the CES‐D score. The CES‐D is a 20‐item measure to rate how often over the past week patients experienced symptoms associated with depression, with response options ranging from 0 to 3 for each item. Scores range from 0 to 60, with high scores indicating greater depressive symptoms [22].

### Statistical analysis

Continuous data were expressed as mean ± standard deviation, while categorical variables were expressed as proportions or percentages. The Shapiro–Wilk test was used to assess the normality of continuous variables. Analysis of variance (ANOVA) was performed to evaluate differences between groups for continuous, normally distributed and homoscedastic data. Otherwise, the Mann–Whitney test was used. Spearman's rank correlation was applied to assess correlations between scores and continuous data. Fisher's exact test was conducted to examine relationships between grouped variables. Kaplan–Meier survival analysis was performed to assess survival to failure (progression to TKA). The log‐rank test was used to evaluate the influence of categorical variables on survival, whereas Cox regression was used to assess the influence of continuous variables on survival. For all tests, a *p*‐value of <0.05 was considered statistically significant. The statistical analysis was performed using SPSS v.19.0 (IBM Corp.).

## RESULTS

According to the search process and previously outlined criteria, a total of 912 patients were identified from the OAI database for inclusion in the analysis. The overall mean age was 61.2 ± 9 years, with a mean BMI of 30.1 ± 5.3 kg/m². Of the total cohort, 62.2% were women and 37.8% were men. At the 9‐year follow‐up, 209 patients (22.9%) progressed to TKA, while 703 (77.1%) did not.

Among patients who did not progress to TKA, the mean age was 62.6 ± 8.5 years and the mean BMI was 30.8 ± 5.1 kg/m². In this group, 63.0% were women and 37.0% were men. The KL grade distribution was as follows: 63.8% had KL grade 2, 29.2% had KL grade 3 and 7.0% had KL grade 4.

Among those who underwent TKA, the mean age was 61.5 ± 8.9 years and the mean BMI was 30.2 ± 5.2 kg/m². In this group, 56.6% were women and 43.4% were men. The KL grade distribution was as follows: 40.2% had a KL grade of 2, 38.7% had a KL grade 3 and 21.1% had a KL grade 4.

The characteristics of the included patients are presented in Table 1.

**Table 1:** Characteristics of the included patents.

Patients	No progression to TKA (*N* = 703)	Progression to TKA (*N* = 209)	Total (*N* = 912)
Women	443	124	567
Men	260	85	345
Age (years)	61.2 ± 9	62.6 ± 8.5	61.5 ± 8.9
BMI (kg/m^2^)	30.1 ± 5.3	30.8 ± 5.1	30.2 ± 5.2
Side R/L	527/176	127/82	654/258
KL 2	449	84	533
KL 3	205	81	286
KL 4	49	44	93

*Note*: Data are displayed as mean ± standard deviation.

Abbreviations: BMI, body mass index; KL, Kellgren–Lawrence; L, left; R, right; TKA, total knee arthroplasty.

The statistical analysis revealed no significant difference of CES‐D score between patients who progressed to TKA and patients who did not. A sub‐analysis was conducted to evaluate whether the CES‐D score influenced progression to TKA based on the KL OA severity, considering that 15.8% of KL 2, 28.3% of KL 3 and 47.3% of KL 4 patients progressed to TKA at 9 years. In patients with KL grade 2, patients who progressed to TKA had a significantly higher CES‐D score compared to patients who did not (6.9 ± 6.6 vs 5.4 ± 5.2, *p* = 0.018). In patients with KL grades 3 and 4, no significant differences of CES‐D score were observed between patients who progressed to TKA and patients who did not.

The survival analysis using Cox regression stratified by KL grade showed a significant association between the CES‐D score and the risk of undergoing TKA in patients with KL grade 2 (hazard ratio [HR] = 1.042, 95% confidence interval [CI]: 1.008–1.076, *p* = 0.015). In patients with KL grade 3 and 4, the association was not statistically significant.

**Figure 1:**
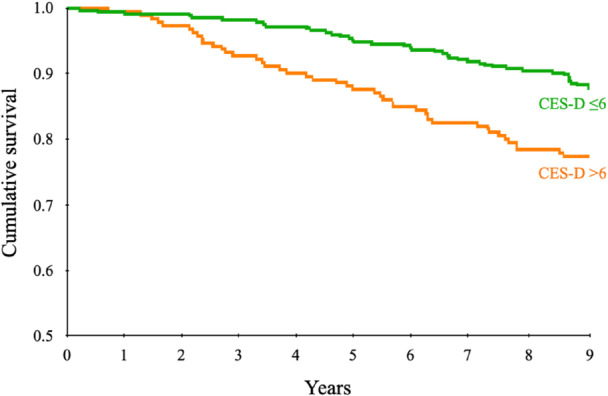
Mantel‐Cox curve illustrating the cumulative survival to total knee arthroplasty over the 9‐year follow‐up period of the Kellgren–Lawrence grade 2 patients based on the Center for Epidemiologic Studies Depression Scale (CES‐D).

Two subgroups were identified based on CES‐D score within each KL category according to a cut‐off of 6 points: patients with CES‐D ≤ 6 and patients with CES‐D > 6. This threshold was determined statistically through exploratory subgroup analyses aimed at identifying potential interactions between depressive symptoms and the risk of undergoing TKA. Specifically, various stratifications were tested based on the available variables extracted for the analysis, but only KL grade showed a significant interaction with CES‐D. Among KL 2 patients, a CES‐D cut‐off of >6 effectively discriminated between those at higher versus lower risk of progressing to TKA. This association was confirmed by a pairwise comparison using the log‐rank (Mantel‐Cox) test of KL 2 patients, which showed a statistically significant difference in terms of progression to TKA between the two CES‐D groups (*p* = 0.001), as reported in Figure 1. No significant differences emerged in the same analysis applied to KL 3 or KL 4 patients, and attempts to identify an alternative cut‐off (e.g., CES‐D > 7 in KL 3) did not yield statistically significant results.

## DISCUSSION

The main finding of this study is that even minor psychological symptoms related to the depression spectrum can be correlated to the progression to TKA. However, this effect was detected only in early KL 2 knee OA patients, but not in the more advanced KL 3 and 4 stages of the disease.

Over the years, numerous studies investigated the factors predicting progression to TKA [17]. By focusing on these factors, clinicians can better plan interventions to the patient's specific needs, ensuring that resources are optimally allocated and surgery is reserved for appropriate cases while avoiding premature or unnecessary procedures [28]. A recent study identified several aspects influencing progression to TKA, including knee swelling, clinical symptoms as documented by the VAS pain and the KOOS, and the degree of joint degeneration in terms of KL grade, even if no difference was noted between the most advanced grades, namely KL 3 and 4 [8]. However, the psychological sphere was not explored.

The present study builds upon these findings by investigating another crucial aspect, beyond the mere physical factors, to be considered when managing patients with knee OA who will potentially undergo TKA. Even minor psychological symptoms related to the depression spectrum, well below the threshold of what commonly considered the level at risk for developing clinical depression, significantly correlate with the trajectory towards TKA. Previous studies focused on the impact of both preoperative and postoperative depressive symptoms on subjective function and pain after this surgical procedure, while lacking longitudinal perspective on the previous phase of the disease, and therefore failing to address their influence on the progression to TKA in knee OA patients [19]. The results of this study documented that depressive symptoms have a significant influence on progression to TKA in early knee OA patients with KL 2, while this influence is lost in the more advanced stages of the disease represented by KL 3 and 4 [8]. This may be due to the fact that in advanced knee OA the trajectory to TKA is influenced by other dominant factors such as pain and functional limitation due to more severe joint derangement. Accordingly, depressive symptoms can hardly be considered one of the driving factors in patients with advanced knee OA, while they have a significant impact in the earlier stages of the disease, where they can influence the progression to TKA, as demonstrated by the present study.

Different mechanisms can explain how depressive symptoms affect progression to TKA in knee OA patients. Depression is well‐known to have a relevant impact on pain perception, including OA pain. Pain represents one of the primary reasons why patients with knee OA seek medical attention, and its characteristics, both in terms of intensity and persistence, are strong predictors of the need for TKA [30]. Patients' pain perception, which can be altered in presence of depressive symptoms, play a key role in the decision‐making process for TKA [23]. High pain levels may also suggest the presence of pain catastrophizing, a psychological condition in which patients exhibit heightened sensitivity and negative pain perception [10]. Depression and catastrophizing are closely related and consistently associated with the reported severity of pain, sensitivity to pain, physical disability and poor treatment outcomes [12]. This is associated with an increased risk of persistent pain even after surgery, potentially leading to dissatisfaction and poor postoperative outcomes [13].

The results of the present study are of clinical relevance as they underline the need to comprehensively consider and address multiple aspects of knee OA when managing this condition in clinical practice. It is key to carefully target the aspects influencing the disease course and ultimately progression to TKA, among which depressive symptoms play a relevant role, particularly in patients with KL 2 knee OA. Addressing the factors associated with progression to TKA might help avoid or at least postpone this invasive procedure, especially in the earlier stages of knee OA. In fact, TKA is increasingly performed not only in elderly patients with end‐stage OA but also in younger and more active adults with milder OA severity [11, 25]. In this light, identifying and addressing modifiable factors, like depressive symptoms, which have the potential to influence progression to TKA, especially in the earlier stages of knee OA, is of paramount clinical relevance. It may help delay this procedure, with considerable social and economic implications, while ultimately optimising the overall management of knee OA patients in clinical practice.

This study has several limitations that require consideration. First, the analysis was limited to the data available within the OAI database. Second, the OAI cohort has been shown to have a higher self‐reported health status compared to a representative survey of individuals with knee OA [27]. As such, the results of this study may not be generalisable to the entire population of knee OA patients. Third, the retrospective design of the study inherently carries a potential risk of bias. Moreover, the investigation of a complex aspect like depressive symptoms goes beyond the information provided by an individual score like the CES‐D and its possible cut‐offs as the one statistically determined in the analysis of the present study, underscoring the need for future studies to expand these findings and further explore this relevant aspect in patients with knee OA to further elucidate its role in the progression to TKA. In the end, the accent should not be placed on a specific CES‐D cut‐off number, rather on the importance of remembering to consider this psychological aspect also in early stages of knee OA management. Other factors may also influence the results, including psychological, physical, morphological, metabolic factors etc… which limit the strength of the correlation found. Finally, depressive symptoms were investigated only at baseline and they might not always be related to TKA progression. Some patients might have made lifestyle changes, in other cases the KL grade might have progressed in parallel with depressive symptoms while in others not, all aspects that could have influenced the results. Overall, these findings should be interpreted within the broader body of literature investigating the influence of depression and in general psychological aspects for both TKA indication and results. Despite these limitations, this study offered valuable insights on the role of depressive symptoms in influencing progression to TKA in patients with knee OA and can help clinicians to adequately consider and address this aspect with important implications for the overall outcome of TKA candidates, thereby optimising the management of these patients in clinical practice. While the study hypothesis was not confirmed on the overall OA population, this study findings suggest that even minor psychological symptoms can be related to the progression to TKA in KL 2 patients. Future studies should aim at further elucidating the risk factors increasing the likelihood of requiring TKA, help tailor the therapeutic approach to the individual patients' characteristics, and optimise the treatment of patients affected by knee OA in clinical practice.

## CONCLUSION

This study showed that even minor psychological symptoms related to the depression spectrum, well below the threshold of what commonly considered the level at risk for developing clinical depression, significantly correlate with the trajectory towards TKA in knee OA patients. However, this effect was detected only in early KL 2 knee OA patients, but not in the more advanced KL 3 and 4 stages of the disease.

## AUTHOR CONTRIBUTIONS

**Alessandro Bensa**: Conceptualisation; methodology; formal analysis; investigation; visualisation; writing—original draft. **Luca Bianco Prevot**: Methodology; data curation; writing—original draft. **Andrea Piano**: Investigation; writing—original draft; visualisation. **Giuseppe M. Peretti**: Writing—review and editing; supervision. **Giuseppe Filardo**: Conceptualisation; methodology; writing—review and editing; supervision; project administration.

## CONFLICT OF INTEREST STATEMENT

The authors declare no conflicts of interest.

## ETHICS STATEMENT

The OAI study was approved by the institutional review board and all participants signed informed consent forms.

## Data Availability

No additional data were generated for this review. The data are found in the referenced papers. The data that support the findings of this study are available from the corresponding author upon reasonable request.
